# miR-125b Suppresses Proliferation and Invasion by Targeting MCL1 in Gastric Cancer

**DOI:** 10.1155/2015/365273

**Published:** 2015-10-04

**Authors:** Shihua Wu, Feng Liu, Liming Xie, Yaling Peng, Xiaoyuan Lv, Yaping Zhu, Zhiwei Zhang, Xiusheng He

**Affiliations:** ^1^Cancer Research Institute, Key Laboratory of Cancer Cellular and Molecular Pathology of Hunan Provincial University, University of South China, Hengyang 421001, China; ^2^Department of Pathology, Shaoyang Medical College, Shaoyang 422000, China; ^3^Graduate Department, University of South China, Hengyang 421001, China

## Abstract

Understanding the molecular mechanisms underlying gastric cancer progression contributes to the development of novel targeted therapies. In this study, we found that the expression levels of miR-125b were strongly downregulated in gastric cancer and associated with clinical stage and the presence of lymph node metastases. Additionally, miR-125b could independently predict OS and DFS in gastric cancer. We further found that upregulation of miR-125b inhibited the proliferation and metastasis of gastric cancer cells in vitro and in vivo. miR-125b elicits these responses by directly targeting MCL1 (myeloid cell leukemia 1), which results in a marked reduction in MCL1 expression. Transfection of miR-125b sensitizes gastric cancer cells to 5-FU-induced apoptosis. By understanding the function and molecular mechanisms of miR-125b in gastric cancer, we may learn that miR-125b has the therapeutic potential to suppress gastric cancer progression and increase drug sensitivity to gastric cancer.

## 1. Introduction

Despite achieving significant progress in therapeutic strategies, gastric cancer remains the second most frequent cause of global cancer mortality [1, 2]. Understanding the precise molecular mechanisms underlying the development and progression of gastric cancer is urgently needed and can provide the basis for molecular treatment strategies [3]. MicroRNAs (miRNAs) are a group of endogenously expressed, noncoding small RNAs. miRNAs negatively regulate the expression of target mRNAs by suppressing translation or decreasing the stability of mRNAs [4]. It has been found that miRNAs play crucial roles in various biological processes, including development, differentiation, apoptosis, and cell proliferation [5–7]. An increasing number of studies have demonstrated that miRNAs can function as oncogenes or tumor suppressors and that they are often dysregulated in tumors [8, 9]. miR-125b has been identified as a tumor suppressor in many tumors, including bladder cancer, breast cancer, and oral squamous cell carcinoma [10–13]. Fassan et al. reported that both miR-125a-5p and miR-125b levels were significantly downregulated throughout the gastric and esophageal carcinogenic cascades [14]. However, a recent study found that miR-125b promotes cell migration and invasion by targeting the PPP1CA-Rb signal pathway in gastric cancer, resulting in a poor prognosis [15], which means that miR-125b could be regarded as oncogene in gastric cancer. To date, the role of miR-125b in gastric cancer has been undefined.

In this study, we found that miR-125b expression is downregulated in 36 stomach tumor specimens and gastric cell lines. miR-125b expression was detected by in situ hybridization on tissue microarrays, and the association between miR-125b levels and clinicopathological factors and prognoses were analyzed. The results indicated that decreased miR-125b levels correlate with advanced clinical stage lymph node metastases and poor clinical outcomes. Additionally, luciferase assay results confirmed* MCL1* (myeloid cell leukemia 1) as a direct target gene of miR-125b. Ectopic overexpression of miR-125b dramatically repressed proliferation, induced apoptosis in vitro, and suppressed tumorigenicity in vivo. Furthermore, miR-125b increased 5-FU-sensitivity through* MCL1*.

## 2. Materials and Methods

### 2.1. Cell Culture

The gastric epithelial cell line GES-1 was purchased from the Beijing Institute for Cancer Research (Beijing, China). The gastric cancer cell lines MGC-803, BGC-823, MKN-28, SGC-7901, HGC-27, AGS, and MKN-45 were obtained from the American Type Culture Collection (ATCC, Rockville, MD). These cells were maintained at 37°C in a 5% CO_2_ atmosphere in RPMI-1640 medium supplemented with 10% fetal bovine serum, penicillin, and streptomycin (Gibco BRL, NY, USA).

### 2.2. Clinical Samples

All of the tissue samples used in this study were collected from the Hunan Provincial Tumor Hospital (Changsha, Hunan, China). Written informed consent was obtained from all of the study participants [9]. This study was approved by the Ethics Committee of the University of South China Health Authority. The collection and use of tissues followed procedures that are in accordance with ethical standards as formulated in the Helsinki Declaration. Tissue samples from 36 gastric cancer patients were used for quantitative real-time PCR (qRT-PCR) analysis. Resected cancerous tissues (tumor) and paired matched normal gastric tissues (normal) were immediately cut and stored in RNAlater solution (Ambion). The tissue microarrays (TMAs) consisted of 126 cases of gastric carcinomas. All of the data, including age, sex, histological grade, tumor size, invasion depth (T stage), and lymph node metastasis, were obtained from clinical and pathological records.

### 2.3. In Situ Hybridization

Tissue microarray slides were deparaffinized and rehydrated [8]. The miR-125b miRCURY LNA custom detection probe (Exiqon, Vedbaek, Denmark) was used for in situ hybridization (ISH). The sequence 5′-3′ (enhanced with LNA) was UCCCUGAGACCCUAACUUGUGA with digoxigenin (DIG) at the 5′ and 3′ ends. Hybridization, washing, and scanning were carried out according to the manuals and protocols provided by the Exiqon Life Science Department. The intensities of miR-125b staining were scored by 0–4, according to the standards of 0-1 (no staining), 1-2 (weak staining), 2-3 (medium staining), and 3-4 (strong staining). The percentages of miR-125b cells in three representative high-power fields of individual samples were analyzed. Those expression scores were equal to scores of the intensities × the percentages of positive cells, and the maximum was 4 and the minimum was 0. Individual samples were evaluated by at least two pathologists in a blinded manner, and those expression scores greater than or equal to 2 were defined as high expression, less than 2 being low expression.

### 2.4. Overall Survival (OS) and Disease-Free Survival (DFS)

DFS was defined as the interval between surgery and the date of diagnosis of the first recurrence or the date of the last follow-up. OS was calculated from diagnosis to the date of death for any cause, and patients who were alive were censored at date of last follow-up visit.

### 2.5. Bioinformatics

Target prediction was performed by online software Targetscan 6.2.

### 2.6. Quantitative RT-PCR Analysis (qRT-PCR)

Total RNAs were extracted from cells with TRIzol reagent (Invitrogen, Carlsbad, USA). Reverse transcription and qRT-PCR reactions were performed by means of a qSYBR-green-containing PCR kit (Qiagen, Germantown, USA). Fold change was determined as 2^−ΔΔCt^. The Ct is the fractional cycle number at which the fluorescence of each sample passes the fixed threshold. The ΔCt was calculated by subtracting the Ct of snRNA U6 from the Ct of the miRNA of interest. The ΔΔCt was calculated by subtracting the ΔCt of the reference sample (paired nontumourous tissue for surgical samples) from the ΔCt of each sample. The primers for qRT-PCR detection of* MCL1* mRNA (F: TAAGGACAAAACGGGACTGG; R: CCTCTTGCCACTTGCTTTTC) were synthesized by Invitrogen. All qRT-PCR was performed with the Bio-Rad C1000 Multicolor Real-Time PCR Detection System (USA).

### 2.7. Dual Luciferase Reporter Assay and 3′UTR Binding Site Mutagenesis

MGC-803 cells (6 × 10^4^) were seeded in 24-well plates immediately prior to transfection. The pMIR-MCL1 plasmids were transfected into MGC-803 cells using Lipofectamine 2000 (Invitrogen) according to the manufacturer's instructions. We also generated several inserts with deletions of 4 bp from the site of perfect complementarity of the* MCL1* gene using the QIAGEN XL-site directed Mutagenesis Kit (QIAGEN, Valencia, CA). The miR-125b mimics and pMIR-*MCL1* plasmids were cotransfected where indicated. Forty-eight hours after transfection, cells were assayed for both firefly and Renilla luciferase using the dual luciferase glow assay (Promega). Transfection experiments were performed in duplicate and repeated at least three times in independent experiments.

### 2.8. Lentivirus Production and Infection

Lentivirus plasmids were cotransfected with pLP1, pLP2, and pLP/VSVG (Invitrogen) into 293T cells (Invitrogen), and virus-containing supernatants were prepared according to the manufacturer's instructions. For lentiviral infection, cells were incubated with virus-containing supernatants in the presence of 6 *μ*g/mL polybrene. Infected cells were selected in the presence of 2 *μ*g/mL puromycin to generate two paired stable monoclonal cell lines. For infection with the GFP-expressing viruses for miRNA expression, flow cytometry analyses (FacsCalibur, Becton Dickinson) were performed to confirm that 90% of cells were infected.

### 2.9. In Vivo Gastric Tumor Model

Male BALB/c nude mice (4–6 weeks old) were purchased from the Hunan Province Laboratory Animal Co., Ltd. (Changsha, China). All of the animal studies were conducted according to protocols approved by the Institutional Animal Care and Use Committee. Briefly, nude mice were inoculated subcutaneously with either MGC-803-control or MGC-803-miR-125b cells (*n* = 5 per group). The formation and growth of human gastric tumors in the recipients were monitored every four days, and the tumor volumes were estimated by measuring two dimensions of the tumors using a digital caliper in a blinded manner. The animal handling and all experimental procedures were approved by the Animal Ethics Committee of the University of South China. Strict sterility was maintained throughout the procedure.

### 2.10. Cell Invasion Assays

Cells were seeded onto the basement membrane matrix in the insert of a 24-well culture plate (EC matrix, Chemicon, Temecula, CA) and fetal bovine serum was added to the lower chamber as a chemoattractant. After 48 hours, the noninvading cells and EC matrix were gently removed with a cotton swab. Invasive cells located on the lower side of the chamber were stained with crystal violet, counted, and imaged.

### 2.11. Western Blot Analysis

Protein lysates from cells were subjected to sodium dodecyl sulfate polyacrylamide gel electrophoresis (SDS-PAGE) and target proteins were detected with primary antibodies recognizing MCL1 (Santa Cruz, USA), cleaved caspase-3, cleaved PARP, and GAPDH (Cell Signaling), respectively. Following incubation with the appropriate horseradish peroxidase- (HRP-) conjugated secondary antibodies (Jackson ImmunoResearch), protein bands were visualized using enhanced chemiluminescence (ECL) plus western blotting detection reagents and exposed in a Bio Image Intelligent Quantifier 1D.

### 2.12. Statistical Analysis

Data were expressed as the mean ± standard error of the mean (SEM) from at least three independent experiments. Comparisons between the groups were analyzed by the *t*-test and *χ* ^2^ test. All differences were considered statistically significant when *P* ≤ 0.05. Statistical analyses were performed using the SPSS16.0 software.

## 3. Results

### 3.1. miR-125b Is Downregulated in Gastric Cancer

**Figure 1:**
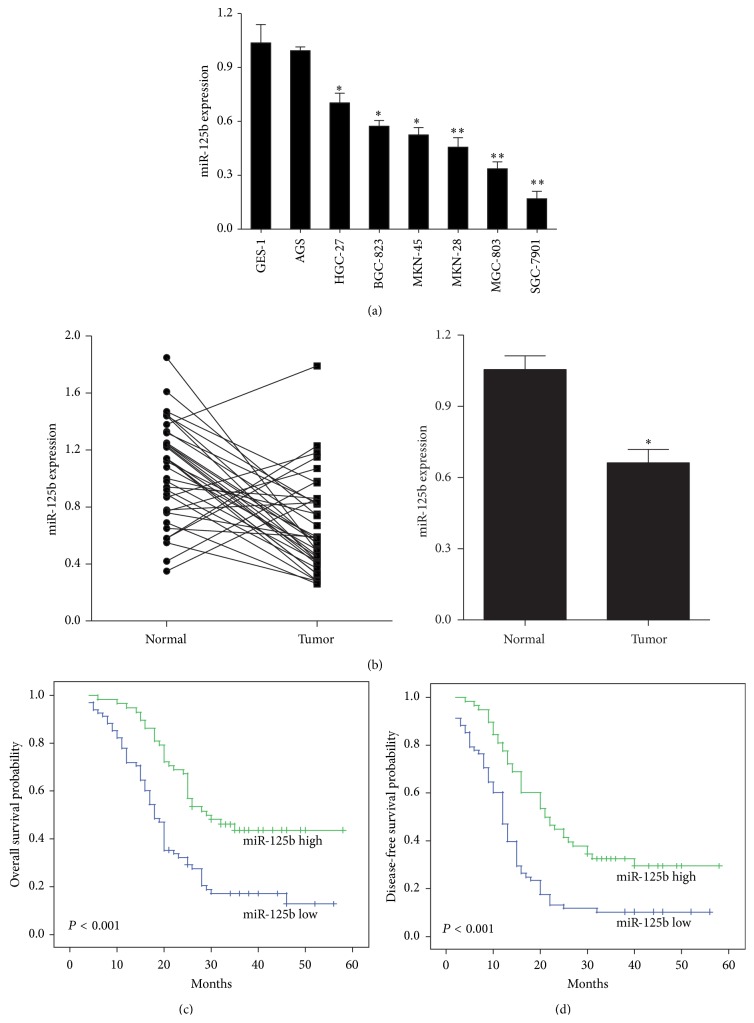
miR-125b expression levels are frequently downregulated in human gastric cancer. (a) Relative expression of miR-125b in 7 cell lines derived from gastric cancer and one nonmalignant gastric cell line (GES-1) was determined by qRT-PCR. The error bars represent the standard deviations (SD) from triplicates of one representative experiment. ^*∗*^ *P* < 0.05 and ^*∗∗*^ *P* < 0.01. (b) miR-125b expression was detected in 36 gastric cancer patient tumors by qRT-PCR. The error bars represent the standard deviations (SD) from triplicates of one representative experiment. ^*∗*^ *P* < 0.05. Survival curves of (c) OS and (d) DFS according to miR-125b expression. Whether miR-125b expression levels were high or low was determined using the Kaplan-Meier method and evaluated using the log-rank test.

First, a series of human gastric cancer cell lines were analyzed to assess the expression profile of miR-125b in gastric cancer using qRT-PCR (Figure 1(a)). Compared with the nonmalignant gastric cell line GES-1, seven of the gastric cancer cell lines showed reduced miR-125b expression, especially the MGC-803 and SGC-7901 cells. We also compared miR-125b expression levels in a series of 36 pairs of gastric cancer tissues and their matched adjacent tissues. Among the 36 gastric cancer patients, significant downregulation of miR-125b was observed in 80.1% of the tumors (29/36, Figure 1(b)), and miR-125b levels decreased (2.5-fold) relative to the adjacent nontumor tissues (Figure 1(b)).

### 3.2. Decreased miR-125b Correlates with Advanced Clinical Stage, Lymph Node Metastases, and Poor Clinical Outcomes

To further verify the results concerning the biological role of miR-125b in gastric cancer, we used in situ hybridization to evaluate miR-125b levels in tissue microarrays (TMAs) consisting of 126 gastric tumor tissues. Our results found that miR-125b levels inversely correlated with invasion depth, clinical stage, and lymph node metastasis (*P* = 0.031, *P* = 0.002, and *P* = 0.001, resp.) (Table 1). However, no significant correlations between miR-125b expression and age, gender, tumor size, or cell differentiation were identified. Our results suggest that miR-125b could play critical roles in progression of gastric cancer. To further examine the significance of miR-125b in terms of clinical prognosis, Kaplan-Meier survival analyses were performed using patient overall survival and relapse-free survival. The results demonstrated that patients with low miR-125b expression had shorter mean months of OS (*P* < 0.001) (Figure 1(c)) and DFS (*P* < 0.001) (Figure 1(d)) than patients with high miR-125b expression.

### 3.3. *MCL1* Is a Target of miR-125b in Breast Cancer Cells

**Figure 2:**
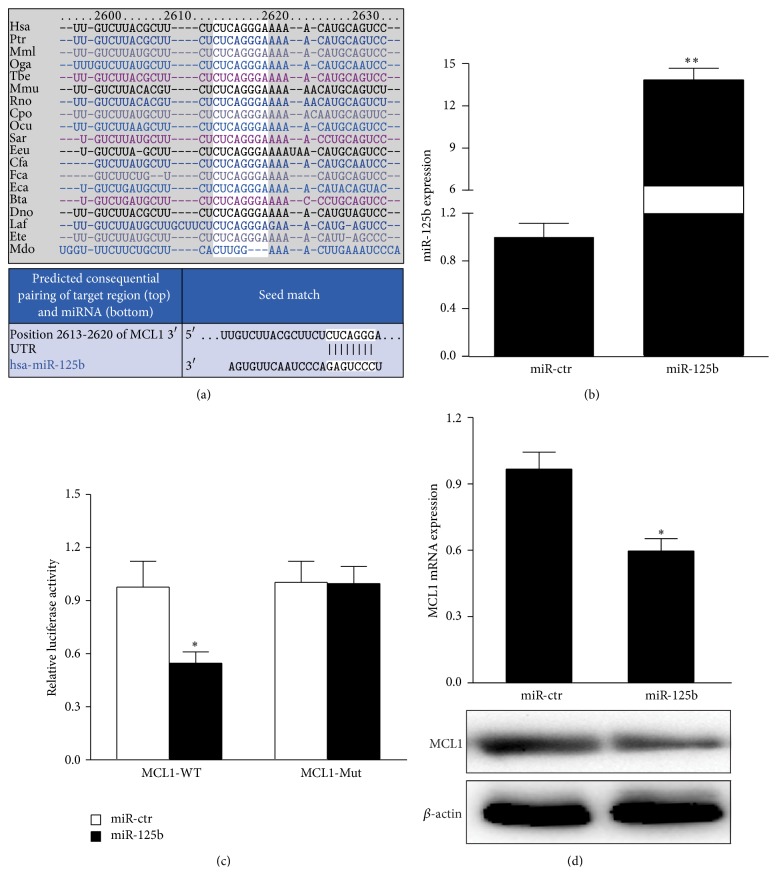
*MCL1* is a target of miR-125b in breast cancer cells. (a) Schematic of the predicted miR-125b site in the 3′UTR of* MCL1* mRNA, which is broadly conserved among vertebrates. (b) miR-125b expression was detected in MGC-803 cells transfected with either miR-125b mimics or the miR-ctr control by qRT-PCR. The error bars represent the standard deviations (SD) from triplicates of one representative experiment. ^*∗∗*^ *P* < 0.01. (c) Luciferase reporter assays were performed after transfection of the indicated pMIR-Report plasmids, a Renilla transfection control plasmid, and with either miR-34a or the relevant scrambled controls. The data shown are the means ± SD of three replicates and are representative of three independent experiments. ^*∗*^ *P* < 0.05. (d) MCL1 mRNA and protein expression levels were much lower in MGC-803 cells transfected with miR-125b mimics compared to cells transfected with miR-ctr. The data shown are the means ± SD of three replicates and are representative of three independent experiments. ^*∗*^ *P* < 0.05.

miR-125 target sites were predicted using online software Targetscan 6.2. The algorithm predicted* MCL1* from the candidate target genes (Figure 2(a)). miR-125b mimics, but not miR-ctr, specifically decreased luciferase expression in the* MCL1*-wt reporter cells. In contrast, no change in relative luciferase expression was observed in cells transfected with the* MCL1*-mut reporter (Figure 2(b)). These results suggest that* MCL1* is a direct target gene of miR-125b. The results from qPCR and western blots showed that enhanced expression of miR-125b by miR-125b mimics in the MGC-803 cells leads to downregulation of endogenous* MCL1* mRNAs and decreased protein levels (Figures 2(c) and 2(d)). Taken together, these results indicate that MCL1 is a direct target gene of miR-125b and can be negatively regulated by miR-125b.

### 3.4. miR-125b Represses Gastric Cancer Progression

**Figure 3:**
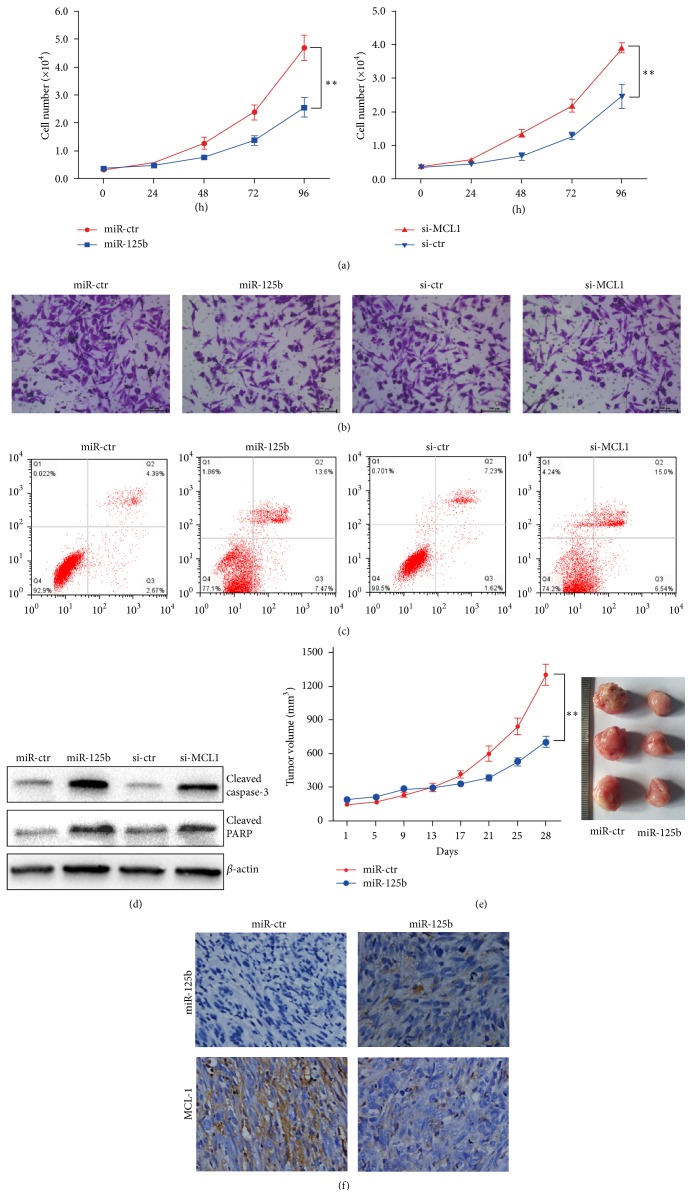
miR-125b represses gastric cancer progression. (a) MGC-803 cells transfected with either miR-125b or miR-ctr (left) and either si-*MCL1* or si-ctr were seeded in 12-well plates at the desired cell concentrations and maintained in medium containing 10% FBS. The cells were counted at the indicated time points in triplicate and their growth rates were recorded. ^*∗∗*^ *P* < 0.01. (b) The invasion assay of the MGC-803 cells transfected with either miR-125b or miR-ctr and either si-*MCL1* or si-ctr. (c) MGC-803 cells were transfected with either miR-125b, miR-ctr, si-*MCL1*, or si-ctr. The apoptotic cells were evaluated with Annexin V-FITC and propidium iodine staining and analyzed by FACS. (d) MGC-803 cells were transfected with either miR-125b, miR-ctr, si-*MCL1*, or si-ctr. The levels of cleaved caspase-3 and cleaved PRAP were evaluated by western blot analysis. (e) MGC-803 cells were subcutaneously injected into nude mice. Then, the effect of an intratumoral injection of 40 *μ*L of either miR-ctr or miR-125b mimic in PBS on tumor volume was examined. Average tumor volumes are shown (*n* = 5 for both experimental groups) from the first injection and continue until after the mice were killed at 28 days (left). After 32 days, the mice were euthanized, necropsies were performed, and tumors were weighed. All data are shown as the mean ± SEM, ^*∗∗*^ *P* < 0.01 (right). (f) In situ hybridization was used to detect the expression of miR-125b, and immunohistochemistry was used to detect the expression of MCL1 in transplanted tumor tissues injected with either miR-125b mimics or the control.

While exploring the functional effect of miR-125b and MCL1 on gastric cancer by cell proliferation, we found that upregulation of miR-125b inhibited the proliferation capacity of MGC-803 cells via functional downregulation of MCL1 expression (Figure 3(a)). The rate of cell survival was considerably lower in cells transfected with miR-125b mimics or* MCL1* siRNA compared to the respective controls. These results indicated that either transfection of miR-125b or knockdown of* MCL1* significantly suppressed gastric cancer cell proliferation in vitro. Furthermore, we found that overexpression of miR-125b or knockdown of* MCL1* significantly inhibited the invasion capacity of MGC-803 cells (Figure 3(b)). Flow cytometry was performed to assess whether this effect was mediated through the induction of apoptosis. The apoptosis rate of MGC-803 cells was increased by transfection with miR-125b mimics or MCL1 siRNA (Figure 3(c)). Western blot analysis of miR-125b mimics/*MCL1*-siRNA-transfected gastric cancer cells indicated a higher expression of cleaved caspase-3 and PARP, which coincide with apoptosis, compared to controls (Figure 3(d)). These data indicate that miR-125b not only inhibited proliferation and invasion but also induced gastric cancer cell apoptosis by directly targeting* MCL1*. To directly evaluate the role of miR-125b in tumour formation and growth in vivo, the xenograft model of human MGC-803 cells in nude mice was adopted. MGC-803 cells infected with miR-125b or miR-ctr lentivirus were injected subcutaneously into each nude mice. After the cells were injected, the tumour volume was monitored every four days. Twenty-eight days after injection, the mean volume and weight of the tumors generated from the MGC-803 cells treated with the miR-125b mimics were significantly lower than those of tumors from mice in the control groups (Figure 3(e)). miR-125b was able to inhibit the expression of MCL1 in vivo (Figure 3(f)). These observations provide strong evidence that overexpression of miR-125b significantly inhibits gastric cancer proliferation in vitro and in vivo.

### 3.5. Transfection of miR-125b Sensitizes Gastric Cancer Cells to 5-FU-Induced Apoptosis

**Figure 4:**
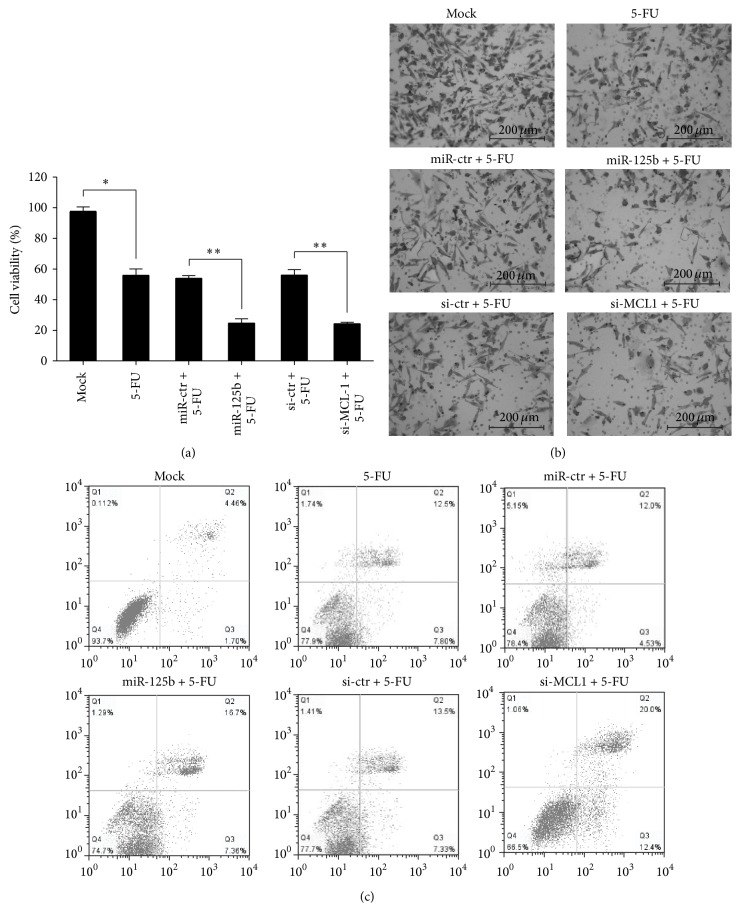
miR-125b inhibited the gastric cancer invasion ability in vitro by targeting Rhoc. (a) MGC-803 were transfected with 5-FU, miR-125b mimics, si-*MCL1*, or combinations of the reagents. MTT assays were performed in MGC-803 cells 48 h after treatment. miR-125b, miR-ctr, si-*MCL1*, or si-ctr was transiently transfected at a concentration of 40 nM. The work concentration of 5-FU was 10 ng/mL. All data are shown as the mean ± SEM, ^*∗*^ *P* < 0.05, ^*∗∗*^ *P* < 0.01. (b) MGC-803 were transfected with 5-FU, miR-125b mimics, si-*MCL1*, or combinations of the reagents. Invasion assays were evaluated by the Transwell assay. (c) MGC-803 were transfected with 5-FU, miR-125b mimics, si-*MCL1*, or combinations of the reagents. The apoptotic cells were evaluated by Annexin V-FITC and propidium iodine staining and analyzed with FACS.

Novel cancer treatment strategies are often composed of conventional chemotherapies and biotherapies, and increasing amounts of evidence indicate that miRNAs are associated with sensitivity to chemotherapeutic drugs, such as 5-fluorouracil in various cancer types. To further assess the synergistic antitumor effects of miR-125b or decreased* MCL1* expression, MGC-803 cells were treated with 5-FU (10 ng/mL) combined with overexpression of miR-125b or* MCL1* silencing. MGC-803 cells with enhanced expression of miR-125b or decreased expression of* MCL1* exhibited greater inhibition of cell proliferation (Figure 4(a)), invasion (Figure 4(b)), and an increase in apoptotic rate (Figure 4(c)) after Taxol treatment. These results suggest that miR-125b is able to sensitize gastric cancer cells to 5-FU-induced apoptosis by targeting* MCL1*.

## 4. Discussion

MicroRNA represents approximately 1% of the genome in different species, each of which has hundreds of different conserved or nonconserved targets, making them key players in various cellular processes [16]. Thus, we believe that more effort should be made to identify relevant miRNAs and to understand the specific mechanisms by which they accomplish their specific functions, particularly their role in the oncogenesis of different tumor types [17–19]. In this study, we used qRT-PCR and ISH to show that miR-125b was frequently downregulated in gastric cancers. Furthermore, we found that miR-125b levels inversely correlated with invasion depth, clinical stage, and lymph node metastasis, suggesting that low expression of miR-125b is associated with gastric cancer progression. Kaplan-Meier survival analyses revealed that patients whose primary tumors displayed a low expression of miR-125b had a shorter OS and DFS in gastric cancer. Further studies showed that overexpression of miR-125b suppressed proliferation and promoted apoptosis capacity in MGC-803 cells. The data from this study suggests that the miR-125b is important for gastric cancer initiation and progression.

We next explored the possible targets of miR-125b in gastric cancer using different computational algorithms. In silico analysis revealed* MCL1* as a candidate target of miR-125b. MCL1 (myeloid cell leukemia 1), a prosurvival member of the Bcl-2 family, was expected to be important due to the association between the aberrant expression of prosurvival Bcl-2 family proteins, tumorigenesis, and resistance to chemotherapeutics [20]. MCL1 is overexpressed in glioma cells. Downregulation of* MCL1* promotes temozolomide-induced apoptosis in gliomas [21]. It has also been demonstrated that several miRNAs induce apoptosis by targeting MCL1 in acute myeloid leukemia [22], lung cancer [23], breast cancer [24], and ovarian cancer [25]. In our study,* MCL1* was further confirmed to be a direct target of miR-125b via luciferase activity assays in gastric cancer cells. We showed that overexpression of miR-125b or downregulation of MCL1 significantly inhibited proliferation and invasion and induced apoptosis in vitro. Due to the heterogeneity and complexity of the mechanisms of tumor progression, it is necessary to develop a new method for modeling the integrated action of these complex relationships and their impact on cancer [26].

Apoptosis is involved in progression and it has been reported that miRNAs play important roles in inducing apoptosis [27] and sensitizing tumor cells to chemotherapeutic agents [28]. In our previous study, we showed that miR-124 could sensitize human gastric cancer cells to 5-FU-induced apoptosis by downregulating EZH2 expression [3]. In this study, we demonstrated that overexpression of miR-125b upregulated apoptosis-related cleaved caspase-3 and PARP and that downregulating MCL1 could enhance the expression of 5-FU-induced cleaved caspase-3 and PARP in gastric cancer cells. These results highlight the ability of miR-125b transfection to increase chemotherapeutic drug-induced apoptosis in gastric cancer.

In summary, we observed downregulation of miR-125b in gastric cancer cells and tissues. We further found that miR-125b is an important tumor suppressor miRNA capable of inhibiting cell proliferation and invasion and promoting cell apoptosis by targeting MCL1 in gastric cancer. Our findings demonstrate that the miR-125b is important for gastric cancer initiation and progression and can be used as a potential therapeutic to suppress gastric cancer proliferation and invasion.

## Figures and Tables

**Table 1:** Analysis of the correlation between expression of miR-125b in primary gastric cancer and its clinicopathological parameters.

Viable	Cases	miR-125b		
		Low	High	*P* value
Age (years)				
<60	73	40	33	0.858
≥60	53	28	25	
Gender				
Male	70	35	35	0.370
Female	56	33	23	
Histological grade				
Well and moderate	32	22	10	0.065
Poor and other	94	46	48	
T stage				
T1-T2	71	32	39	0.031
T3-T4	55	36	19	
TNM stage				
I-II	51	19	32	0.002
III-IV	75	49	26	
Lymph node metastasis				
Present	88	56	32	0.001
Absent	38	12	26	
